# Research for Health Care and Policy on HIV/AIDS: proceedings of the third edition of the Cameroon HIV Research (CAM-HERO) 2022, Kribi, Cameroon, from 1^st^ to 3^rd^ December 2022

**DOI:** 10.11604/pamj.2023.46.6.40711

**Published:** 2023-09-06

**Authors:** Anastase Dzudie, Patrice Tchendjou, Eveline Mboh Khan, Rogers Ajeh, Friedrich Thienemann, Appolinaire Tiam, Boris Tchounga, Joseph Fokam, Clement Ndongmo, Peter Vanes Ebasone, Gabriel Mabou, Andre Pascal Goura, Emile Nforbih Shu, Tshimwanga Katayi, Pascal Atanga Nji, Marc Lionel Ngamani, Clarisse Lengouh, Felicite Naah Tabala, Ebako Ndip Takem, Albert Bakor, Lorraine Guedem Nekame, Tatiana Djikeussi, Ezechiel Ngoufack Jagni Semengue, Kandel Tebong Fon, Collins Chenwi, Judith Lainsi Nasah, George Njie Ngeke, Masha Roland Tasha, Nicoline Ndiforkwah, Grace Nyemb, Emmanuel Nshom, Clovis N´Draman, Charlotte Ayima Wenze, Leonie Simo, Gilles Ndayisaba, Walters Kum Kang, Alex Durand Nka, Boris Youngui Tchakounte, Simplice Lekeumo, Esther Neba, Nyenty Agbornkwai, Therese Abong Bwemba, Pius Tih Muffih, Anne Cecile Zoung-Kany Bisseck, Louis Richard Njock

**Affiliations:** 1 Clinical Research Education, Networking and Consultancy, Yaoundé, Cameroon,; 2 Faculty of Medicine and Biomedical Sciences, University of Yaoundé I, Yaoundé, Cameroon,; 3 Department of Internal Medicine and Subspecialties, Douala General Hospital, Douala, Cameroon,; 4 Lown Scholars Program, Department of Global Health and Population, Harvard T H Chan School of Public Health, Boston, United States of America,; 5 Elizabeth Glaser Pediatric AIDS Foundation (EGPAF), Yaoundé, Cameroon,; 6 Cameroon Baptist Convention Health Services (CBCHS), Bamenda, Cameroon,; 7 National AIDS Control Committee, Ministry of Public Health, Yaoundé, Cameroon,; 8 General Medicine & Global Health, Department of Medicine and Cape Heart Institute, Faculty of Health Science, University of Cape Town, Cape Town, South Africa,; 9 Department of Internal Medicine, University Hospital Zurich, University of Zurich, Zurich, Switzerland,; 10 Elizabeth Glaser Pediatric AIDS Foundation, Washington DC, USA,; 11 Chantal BIYA International Reference Centre for research on HIV/AIDS Prevention and Management, Yaounde, Cameroon,; 12 Faculty of Health Sciences, University of Buea, Buea, Cameroon,; 13 National HIV Drug Resistance Prevention and Surveillance Working Group, Ministry of Public Health, Yaounde, Cameroon,; 14 Division of Global HIV and TB, Center for Global Health, U.S, Center for Diseases Control and Prevention, Yaoundé, Cameroon,; 15 Department of Medicine, Faculty of Health Science, University of Cape Town, Cape Town, South Africa,; 16 Division of Health Operational Research, Ministry of Public Health, Yaoundé, Cameroon,; 17 Bamenda Regional Hospital, Bamenda, Cameroon,; 18 National Ethics Committee, Yaoundé, Cameroon,; 19 Catholic University of Central Africa, School of Health Sciences, Yaounde, Cameroon,; 20 General Secretariat, Ministry of Public Health, Yaoundé, Cameroon

**Keywords:** HIV, research, Cameroon, meeting report

## Abstract

Cameroon is committed to reaching HIV epidemic control through coordinated efforts by the Ministry of Public Health, the National AIDS Control Committee, bilateral/multilateral institutions and implementing partners. The third edition of the Cameroon HIV Research Forum (CAM-HERO) was held in Kribi from December 1^st^ to 3^rd^, 2022, with the theme “Research for Health Care and Policy on HIV/AIDS.” The conference brought together local and international scientists and clinicians, policymakers, and regulatory authorities to 1) disseminate HIV research findings and HIV policy; 2) foster operational research collaboration; 3) build research capacity through training on basics of research methods and CAM-HERO young investigator Awards; and 4) initiate a guideline for promoting HIV/AIDS research in Cameroon. The main activities included training on research methodology and basic principles in bioethics, presentations of selected abstracts, and awards for top research. A total of 35 abstracts (16 oral presentations, 16 posters, and 3 late-breaker-abstracts) were selected for presentation following a rigorous review. The conference ended with evidence-based recommendations and a way-forward statement for the development of a National Guide for HIV/AIDS research in Cameroon, with the aim of improving the quality and quantity of research agenda and projects nationwide.

## Conference Proceedings

The Cameroon HIV/AIDS Operational Research Forum (CAM-HERO) is an initiative aimed at improving and optimizing relevance, performance, and translation of HIV research evidence to argument policy making and practice on HIV care in Cameroon. The main goal of CAM-HERO is to contribute to accelerating the achievement of the UNAIDS fast-track targets 95-95-95 in Cameroon by working on several key objectives. Specifically, to: 1) Build capacity in HIV research 2) Identify new, relevant and priority research questions through improved collaboration with the Ministry of Public Health, 3) Foster operational research collaboration among research organizations, and 4) Disseminate research findings and facilitate their translation into policy. After the 2020 and 2021 successful editions [1,2], and the resulting five research priorities and corresponding research approaches [3], the 2022 edition of CAM-HERO was held. It took place from December 1-3 at the Hotel le Lagon Resort in Kribi. The theme of this edition was “**Research for Health Care and Policy on HIV/AIDS**”. This third edition brought together scientists, clinicians, policymakers, program managers, research regulatory authorities and representatives from local and international research institutions. A total of 65 abstracts were submitted for review, out of which 35 were retained, 16 for oral presentation, 16 for poster presentations, and 3 as late breaker-presentations. Participants were trained on research methodology and basic principles in bioethics on the first day. Day two and three saw the presentation of abstract and plenary sessions and the closing ceremony. These sessions were enriched with discussions arising from outstanding contributions of the various participants and speakers, under the leadership of the Ministry of Public Health of Cameroon.

**Figure 1:**
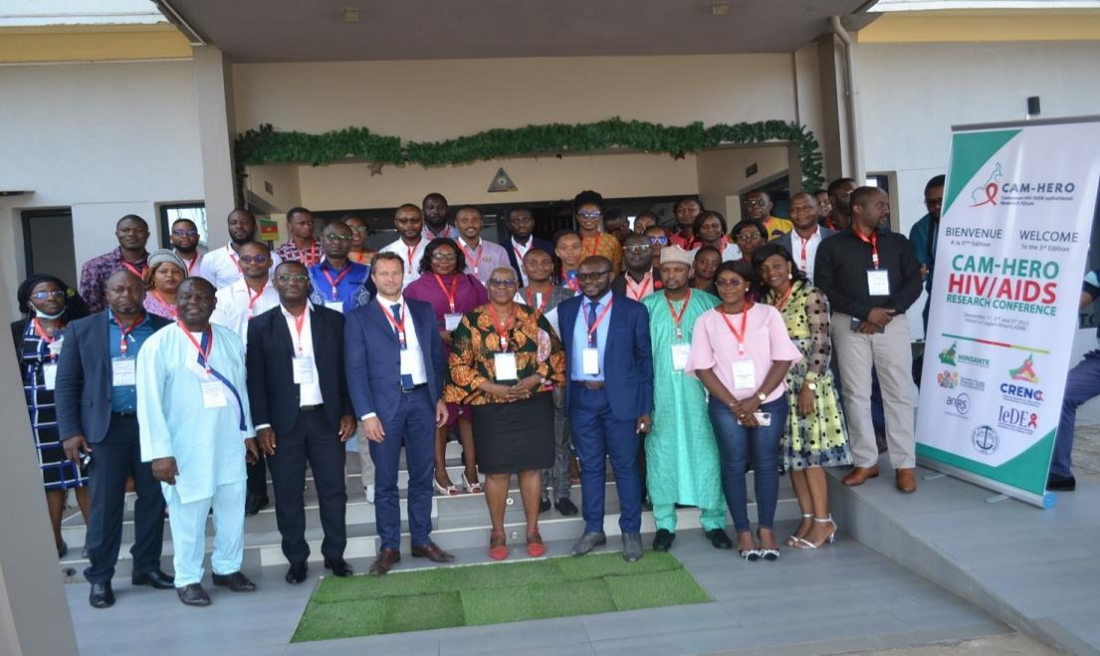
speakers, chairs, and participants of the first day of the research methodology training

**Day 1: December 1^st^, 2022:** the 3^rd^ edition of CAM-HERO was launched with Research Methodology and Bioethics Training. The sessions were chaired by Professor Anne-Cécile Zoung-Kanyi Bissek, Head of the Division of Health Operational Research (DROS), Ministry of Health (DROS/ MoH) and facilitated by Professor Friedrich Thienemann of the University of Cape Town, South Africa (UCT, SA) and University of Zurich, Switzerland (UZH, CH), Dr Patrice Tchendjou of the Elizabeth Glaser Pediatric AIDS Foundation (EGPAF), Dr Appolinaire Tiam (EGPAF) and Professor Anastase Dzudie of the Clinical Research Education Network and Consultancy - International epidemiologic Databases to Evaluate AIDS (CRENC-Ie DEA). The team took family pictures (Figure 1) before starting with the training sessions.

**Session 1: research questions and design:** the session aimed to build capacity of the participants on the fundamentals of a research question, hypothesis generation and design. Professor Thienemann covered the process of developing a research question using the Patient/Population, Intervention/Indicator, Compare/Control, and Outcome (PICO) model. Professor Dzudie explored study design, including types and selection criteria, and the differences between qualitative and quantitative approaches. Dr Tchendjou provided practical examples of questions and designs illustrating the previous talks using the Population Exposure Outcome (PEO) models. The session emphasized the importance to contribute to improvements in health, social welfare, and poverty reduction through domestic production and application of research findings.

**Session 2: research design and strategy in HIV/AIDS:** Professor Thienemann introduced Randomized Clinical Trials (RCTs), including the different phases, as well as ethical and regulatory principles, such as participant enrolment, informed consent, confidentiality, compensation, adverse events, and the roles of those involved. Dr Tiam presented the stepped wedge cluster randomized trial design, a novel research study design to evaluate service delivery type of interventions. This design involves random and sequential crossover of clusters from control to intervention until all clusters are exposed. It is a pragmatic study design which can reconcile the need for robust evaluations with political or logistical constraints that are constant in low- and middle-income countries including Cameroon.

**Session 3: introduction to statistics and scientific writing:** Professor Dzudie presented on Descriptive Statistics, covering variables, types and hierarchy, purposes of analysis, scales of measurement, population distribution, probability distributions, and statistical methods. Dr Tiam discussed Scientific Styles and IMRAD Structure, defining a conference abstract and its key components. Participants were encouraged to practice abstract writing for future conferences. At the end of the training a post-test was taken, and certificates were awarded to participants. A Good Clinical Practice (GCP) course was recommended for future CAM-HERO research training.

**Figure 2:**
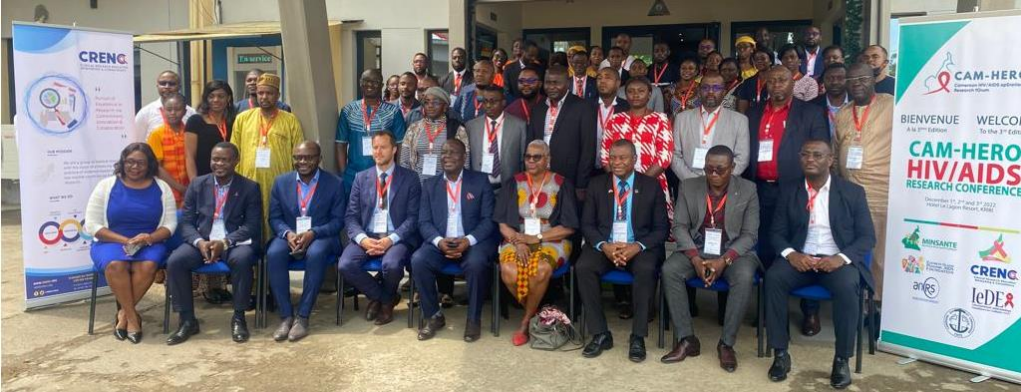
speakers, chairs, and participants of the second day of the CAM-HERO conference 2022

**Day 2: December 2^nd^, 2022:** the second day of the event was marked by abstract sessions and plenary discussions. The sessions were chaired by Professor Anne-Cécile Zoung-Kanyi Bissek of the Ministry of Public Health, Professor Pius Tih of the Cameroon Baptist Health Services (CBCHS), Dr Clement Ndongmo of the Centres for Disease Control and Prevention (CDC - Cameroon), Dr Edouard Tshimwanga (CBCHS), Professor Friedrich Thienemann (UCT, SA & UZH, CH) and Dr Pascal Atanga (CBCHS). Mrs Felicite Naah and Dr Therese Abong presented on administrative and ethical procedures regarding human subject research in Cameroon, and the new Cameroon research ethics law. The goal of these presentations was to create awareness on existing research regulations concerning health research involving human subjects and the implication of these regulations to researchers. Recommendations from this discussion included improving collaboration with ethics committees, setting-up an online system for protocol submission and reporting, establishing Research Ethics Committees (REC) in all regions as well as institutional review boards (IRB) in major research and academic institutions of the country, facilitating collaboration between the ethics committee and researchers, and establishing a yearly calendar for researchers to prepare & monitor submissions. Dr Rogers Ajeh, the Deputy Permanent Secretary, National AIDS Control Committee (NACC) discussed NACC´s research mission, organization, and collaboration goals. He also made a presentation on epidemic preparedness and response: lessons from COVID-19 associated HIV service delivery disruptions. Professor Anastase Dzudie closed this part of the meeting with a guided discussion on the HIV/AIDS and Non-Communicable Diseases (NCDs) comorbidities in Africa. He demonstrated the interaction between HIV and NCDs, showed the trends over the past two decades and called for systematic research towards strategic interventions. A multi-stakeholder approach for an integrated management of care was recommended, with researchers, clinicians, civil societies and the government taking leadership. Simple steps for a start would include 1) Screening and reporting, 2) Basic equipment, 3) Protocols for case-finding approach, 4) Essential drug supplies, 5) Training of staff, monitoring and review. The team took family pictures (Figure 2) and the meeting ended for the second day.

**Day 3: December 3^rd^, 2022:** the last day of CAM-HERO 2022 featured a discussion on the National HIV/AIDS Research Agenda, poster presentations, and the CAM-HERO Awards of Excellence.

**The national HIV/AIDS research agenda:** the presentations of this session were chaired by Professor Anne Bissek and Dr Appolinaire Tiam. Experts offered the following presentations to enrich deliberations on the National HIV/AIDS research agenda: “The situation of HIV/AIDS research in Cameroon: A review of published literature from 2002 to 2022” by Dr Clement Ndongmo and Dr Takem Ebako (CDC-Cameroon). The speaker demonstrated that in Cameroon, research priorities could be geared towards HIV testing, treatment, and virological outcomes, amongst others including differentiated service delivery strategies and use of dolutegravir (DTG)-based regimen in adults and children. Dr Joseph Fokam (CIRCB) presented on “Recent developments on HIV drugs resistance in Cameroon”. Major HIV drug resistance findings were unveiled with the resistance and clinical monitoring of DTG-uptake in Cameroon; the effectiveness of DTG in Sub-Saharan Africa was covered, future drugs and resistances were demonstrated, and he concluded with the priority research on HIV drug resistance. Professor Friedrich Thienemann presented “Updates on HIV/AIDS clinical trials in Africa”. There is a rise in HIV clinical trials conducted in Africa and one of the recent studies investigated the effectiveness of Dolutegravir in ART regimens. These trials have shown that, people living with HIV (PLHIV) who started tenofovir/lamivudine/dolutegravir (TLD) regimen (who never failed a prior regimen) had a very low risk of developing DTG resistance meanwhile those who failed a prior regimen and then transitioned to TLD had 1-3% risk of DTG resistance. All presentations were accompanied by enthusiastic interactions. Key points arising were: 1) The need for inclusion of experts across different fields, 2) Creating a resistance monitoring program and a pharmacovigilance consortium, 3) Implications of pharmaceutical firms in future studies. This session was closed by Professor Dzudie who made a presentation on “What are the objectives of a Cameroon HIV/AIDS Research Agenda”. The overall goal of this Agenda is to guide operational research both at the national and the regional level and to support the NACC in its efforts to attain the HIV/AIDS “Treat all” or “Test and Treat” goals. The idea was enthusiastically received, along with ideas for i) Possible sources of funding; ii) Implication of opportunistic infections, vulnerable populations, and different actors; iii) Include and update HIV Research priorities; iv) Collaboration agreements between organizations.

**CAM-HERO Awards:** the day was crowned with the CAM-HERO awards ceremony. It was presided over by the Director of the DROS/MoH, Professor Bissek. She congratulated the CAM-HERO organizers and attendees for their dedication to advancing HIV/AIDS research in Cameroon and for the introduction of the day-1 event on training in research methodology and bioethics. All the participants received Certificates of Participation and a handful of prominent researchers were awarded certificates of achievement in recognition of their tireless dedication to HIV/AIDS research in Cameroon. The award for the first-best oral abstract presentation went to Gabriel Tchatchouang Mabou and the second went to Dr Ezechiel Ngoufack Semengue. The award for the first best poster presentation went to Djomo Nzaddi Audrey Raissa and the second award to Dr Ayima Charlotte Wenze. Special awards of excellence in leadership were offered to Professor Anne Bissek (DROS/MOH), Professor Anastase Dzudie (CRENC/IeDEA), Professor Friedrich Thienemann (UCT, SA & UZH, CH), Professor Pius Tih Muffih (CBCHS), Dr Appolinaire Tiam (EGPAF), Dr Clement Ndongmo (CDC-Cameroon), Dr Joseph Fokam (CIRCB and FHS/UB) and Dr Thérèse Abong (CNERSH).

**Funding:** the third edition of CAM-HERO in Kribi received funding from the Elizabeth Glaser Pediatric AIDS Foundation (EGPAF), the Cameroon Baptist Convention Health Services (CBCHS), and the International Epidemiology Databases to Evaluate AIDS-Clinical Research Education Networking and Consultancy research group (CRENC-IeDEA).

**Disclaimer:** the findings and conclusions in this report are those of the authors and do not necessarily represent the official position of the agencies.

## Conclusion

The third edition of CAM-HERO successfully brought together key stakeholders to support young researchers in strengthening their research skills, discussing national research findings & priorities, refining HIV/AIDS research in Cameroon, and building the next generation of HIV scientists at country-level. It featured workshops on research methodology, ethics, and research administration, priority areas of HIV/AIDS research, as well as opportunities for research collaboration and innovation. Event highlights were the commencement of a process towards the development and adoption of the National Guidelines for HIV/AIDS research in Cameroon, aimed at enhancing the quality of research agenda & projects in the country.
